# Joint Beamforming for Integrated Satellite–Terrestrial ISAC Systems

**DOI:** 10.3390/s26072273

**Published:** 2026-04-07

**Authors:** Tengyu Wang, Qian Wang

**Affiliations:** 1 School of Electrical and Telecommunications Engineering, University of New South Wales, Sydney, NSW 2052, Australia; z5612204@ad.unsw.edu.au; 2 Department of Computer Science, Durham University, Stockton Road, Durham DH1 3LE, UK

**Keywords:** beamforming, integrated satellite–terrestrial networks, millimeter-wave, integrated sensing and communications, robust optimization

## Abstract

Satellite–terrestrial integrated networks provide seamless global coverage, especially in remote areas where terrestrial deployment is costly. Integrated sensing and communications (ISAC) enhances spectral efficiency by merging both functions on a single platform. This paper proposes a novel integrated satellite–terrestrial ISAC architecture, where a satellite performs simultaneous communication and sensing. The satellite transmits communication signals and sensing waveforms to an Earth Station, which then relays them to a terrestrial base station to serve multiple users. We formulate a joint beamforming design problem to maximize the sum rate of users under quality-of-service constraints, backhaul capacity limits, beampattern requirements for sensing, and power budgets. With perfect channel state information, the non-convex problem is transformed into a difference-of-convex form and solved via the convex–concave procedure. For imperfect channel state information, a robust method combining successive convex approximation and the S-procedure is developed. Simulations show the proposed design outperforms benchmarks and is suitable for low-Earth orbit satellite systems.

## 1. Introduction

### 1.1. Related Work and Motivation

Satellite communications have experienced rapid technological evolution in recent years, driven by the large-scale deployment of low-Earth orbit (LEO) constellations and the increasing commercial maturity of small satellite platforms. Operating at altitudes typically between 500 and 2000 km, low-Earth orbit satellites substantially reduce propagation latency and improve link quality compared with geostationary systems, thereby enabling broadband access and real-time services over wide geographic areas. These advantages make low-Earth orbit-based systems particularly attractive for remote, rural, and geographically isolated regions where terrestrial infrastructure deployment is economically prohibitive or logistically infeasible [1,2,3]. Consequently, extensive research efforts have focused on constellation architecture design, satellite access protocols, dynamic spectrum sharing, and interference management in satellite and non-terrestrial networks [2,4,5,6]. Representative studies have investigated digital-twin-enabled lifecycle management for large-scale satellite–terrestrial deployments, dynamic beam hopping and spectrum sharing mechanisms to mitigate co-channel interference between satellite and terrestrial systems [4,5,7], and advanced resource allocation strategies accounting for time-varying coverage, handover dynamics, and Doppler effects in dense low-Earth orbit constellations [1,2]. Despite these advances, the existing satellite communication literature remains fundamentally connectivity-centric, with system objectives primarily centered on throughput, latency, coverage probability, and reliability, while sensing-related functionalities are rarely incorporated as explicit design targets within the network optimization framework.

Beyond standalone satellite systems, the integration of satellite and terrestrial networks has emerged as a key paradigm for building resilient and ubiquitous communication infrastructures. Integrated satellite–terrestrial networks leverage the complementary strengths of satellites, which provide wide-area coverage and broadcast capability, and terrestrial networks, which offer high capacity, low latency, and flexible scheduling [1,8]. Prior research has extensively examined cooperative transmission strategies, joint resource management, and interference coordination in integrated satellite–terrestrial networks operating under shared-spectrum constraints [1,2,9]. Application-oriented studies have demonstrated the effectiveness of satellite–terrestrial integration in challenging scenarios such as maritime communications, where robust beamforming and interference-aware designs are essential [10], as well as in critical infrastructure applications including smart-grid monitoring, emergency communications, and disaster recovery [11]. In particular, advanced beamforming and precoding techniques have become fundamental tools for mitigating cross-tier interference and improving spectral efficiency in such heterogeneous architectures, motivating the exploration of joint beamforming strategies across satellite and terrestrial segments. In parallel, cross-domain network management and orchestration frameworks have been proposed to address the operational complexity of large-scale integrated networks [12], and physical-layer security techniques have been investigated to cope with the increased vulnerability introduced by spectrum sharing across network tiers [13,14,15]. Nevertheless, these works almost exclusively focus on communication-oriented performance metrics, systematically overlooking the potential role of integrated satellite–terrestrial networks in supporting large-scale sensing and monitoring tasks, which are often crucial in remote and infrastructure-scarce regions.

In parallel with the evolution of satellite and integrated networks, integrated sensing and communications (ISAC) has emerged as a promising paradigm for future wireless systems, enabling simultaneous data transmission and environmental sensing using shared spectrum and hardware resources. Early ISAC studies mainly focused on theoretical limits and waveform design, while recent research has shifted toward system-level architectures and practical applications [16,17,18,19]. Standardization activities have also identified various ISAC use cases, including environmental monitoring, public safety, industrial automation, and intelligent transportation systems [20,21].

Recent studies demonstrate that communication signals can support large-scale environmental sensing tasks such as precipitation estimation, flood monitoring, and vegetation observation [22]. Meanwhile, artificial intelligence techniques have been introduced to improve ISAC adaptability and reduce optimization complexity [23,24]. Algorithmic advances include graph neural network-based beamforming learning for cell-free massive MIMO ISAC systems [25,26], multistatic sensing architectures that enhance spatial coverage [27,28], and UAV-assisted ISAC frameworks for blockage-prone or disaster scenarios [29,30,31].

However, most existing ISAC studies remain terrestrial-centric and implicitly assume dense infrastructure and reliable high-capacity backhaul, which may not be available in rural or geographically isolated regions.

To address these limitations, recent work has explored integrating sensing capabilities into satellite and non-terrestrial networks [32]. Existing studies include NTN-enabled ISAC frameworks for wide-area sensing and resource allocation [33,34,35], bistatic and multistatic LEO ISAC architectures to mitigate severe echo path loss [36,37], and prototype systems combining LEO communications with synthetic aperture radar sensing [38]. Additional studies investigate RIS-assisted satellite ISAC [39,40], movable-antenna-enabled LEO sensing [41], and constellation-level cooperation for joint sensing and communications [42].

The self-backhauled satellite–Earth Station–terrestrial base station architecture has been widely adopted in integrated satellite–terrestrial networks due to its flexibility in extending coverage and reducing infrastructure deployment costs. In such architectures, LEO satellites experience significant Doppler shifts due to their high mobility, which must be compensated to maintain reliable feeder links [43]. Satellite feeder links and terrestrial access links typically operate over a shared spectrum, which gives rise to severe cross-tier co-channel interference. Moreover, the satellite-to-Earth Station link serves as a fundamental backhaul bottleneck that jointly constrains both communication throughput and sensing performance [44]. Integrated sensing and communications (ISAC) has emerged as a promising paradigm to enable simultaneous data transmission and environmental sensing using shared spectrum and hardware resources [45]. These challenges necessitate the joint design of transmit beamforming across satellite sensing signals and terrestrial communication signals, such that interference can be effectively managed while satisfying sensing beampattern requirements and backhaul capacity constraints [46]. However, these critical characteristics are not adequately captured in existing ISAC frameworks, which often assume idealized backhaul links or decoupled sensing and communication operations. Consequently, there remains a significant gap in the literature regarding the joint beamforming and resource optimization for practical self-backhauled satellite–terrestrial ISAC systems under realistic interference and backhaul constraints [47,48].

### 1.2. Contributions

To address the above research gap, this paper makes the following key contributions:

- We propose a novel integrated satellite–terrestrial self-backhauled integrated sensing and communications architecture in which an Earth Station and a terrestrial base station collaboratively relay satellite connectivity while jointly supporting communication and sensing functionalities.
- We develop a unified system model that explicitly captures cross-tier co-channel interference between satellite feeder links and terrestrial access links, as well as the fundamental backhaul capacity limitation imposed by the satellite-to-Earth-station link.
- We formulate a joint optimization problem for communication beamforming and sensing covariance design under quality-of-service requirements, sensing beampattern constraints, power budgets, and backhaul capacity constraints.
- We design efficient iterative algorithms for both perfect and imperfect channel state information scenarios, leveraging difference-of-convex programming, successive convex approximation, and the S-procedure, and provide convergence guarantees.
- Numerical results demonstrate that the proposed design significantly outperforms benchmark schemes in both communication throughput and sensing performance while explicitly revealing the impact of backhaul bottlenecks in integrated satellite–terrestrial sensing and communications systems.

## 2. System Model and Problem Formulation

**Figure 1:**
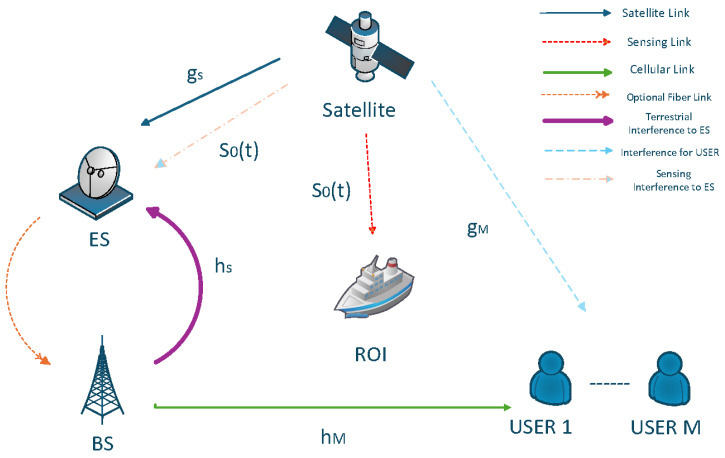
The integrated satellite–terrestrial self-backhauled network.

We consider an integrated satellite–terrestrial network system that jointly performs communication and sensing operations. As illustrated in Figure 1, the proposed ISTS-integrated sensing and communications framework comprises three wireless links operating simultaneously within the same millimeter-wave frequency band: a feeder communication link from the multi-antenna satellite to an Earth Station, multiple terrestrial downlink communication channels from the Earth Station-connected base station to single-antenna user equipment, and a sensing link from the satellite toward a ground region of interest where the satellite transmits a dedicated radar-like sensing waveform to perform detection and localization tasks.

### 2.1. Signal Model

In the proposed IST-ISAC framework, the signal transmission and reception process unfolds as follows, mirroring the flow depicted in Figure 1. The satellite transmits two distinct signals: a communication symbol intended for the Earth Station, denoted by $x_{s}\left(t\right)$ with unit power, and a dedicated sensing waveform $s_{0}\left(t\right)$ that illuminates a ground region of interest. Concurrently, the base station transmits downlink symbols to *M* terrestrial users, with the symbol for user *m* represented by $x_{m}\left(t\right)$, also having unit power.

The satellite applies a beamforming vector v to its communication symbol, while the base station employs a set of beamforming vectors $\left\{w_{m}\right\}$ to serve the multiple users. The sensing waveform $s_{0}\left(t\right)$ has zero mean and its second-order statistics are characterized by the covariance matrix $V_{0}=E\left\{s_{0}\left(t\right)s_{0}^{H}\left(t\right)\right\}$, which is positive semidefinite and shapes the sensing beampattern. The sensing performance is characterized by the beampattern, which represents the spatial distribution of the transmitted power. The beampattern at an angle θ is defined as the expected power radiated in that direction θ.

(1) $$P\left(\theta \right)=a^{H}\left(\theta \right)V_{0}a\left(\theta \right),$$

where a(θ) is the array steering vector of the satellite antenna array toward direction θ, and $V_{0}=E\left\{s_{0}\left(t\right)s_{0}^{H}\left(t\right)\right\}$ is the covariance matrix of the sensing waveform. To ensure effective sensing performance, the beampattern must meet certain requirements. Typically, the beampattern gain at specific sensing directions $\theta_{t}$ (target directions) should exceed a predetermined threshold $\Gamma_{t}$ to guarantee sufficient signal-to-noise ratio for target detection and parameter estimation. This constraint can be expressed as

(2) $$a^{H}\left(\theta_{t}\right)V_{0}a\left(\theta_{t}\right)\geq \Gamma_{t},\;\forall t=1,\ldots ,T,$$

where *T* is the number of sensing targets or directions of interest. The sensing waveform $s_{0}\left(t\right)$ also contributes to the total transmit power of the satellite, which is subject to the power constraint given in Equation (17e). Moreover, the sensing waveform causes interference at both the Earth Station and terrestrial user equipment and must be accounted for in the overall system design.

At the Earth Station, the received signal is a superposition of the desired satellite communication signal, interference from base station transmissions that leak into the Earth Station receiver, the satellite sensing waveform, and additive noise. This composite signal can be expressed as

(3) $$y_{s}\left(t\right)=g_{s}^{H}v\;x_{s}\left(t\right)\;e^{j2\pi f_{s}\left(t\right)t}+\sum_{m=1}^{M}h_{s}^{H}w_{m}\;x_{m}\left(t\right)+g_{s}^{H}s_{0}\left(t\right)+z_{s}\left(t\right).$$

where the first term is the desired satellite communication signal, the second term is the terrestrial interference from the base station (BS) transmissions, the third term constitutes the interference from the satellite’s dedicated sensing waveform, and the final term is the additive white Gaussian noise (AWGN) at the Earth Station (ES) receiver with zero mean and variance $\sigma_{s}^{2}$. Here, $g_{s}$ denotes the channel vector from the satellite to the ES, v is the satellite’s beamforming vector for communication, $x_{s}\left(t\right)$ is the unit-power satellite communication symbol, and $f_{s}\left(t\right)$ is the Doppler shift experienced on the satellite link. The vector $h_{s}$ represents the interference channel from the BS to the ES, $w_{m}$ is the BS beamforming vector for terrestrial user *m*, and $x_{m}\left(t\right)$ is the unit-power downlink symbol for user *m*. The sensing waveform is denoted by $s_{0}\left(t\right)$.

Due to the high mobility of LEO satellites, Doppler shifts can be significant at mmWave/Ka-band frequencies. In practical systems, the dominant Doppler is compensated via ephemeris-based pre-compensation and carrier tracking (e.g., 3GPP NTN [43]). After compensation, the residual Doppler is small and can be absorbed into the effective channel, which justifies the quasi-static narrowband model adopted in this work. This assumption is also consistent with recent studies on Doppler-resilient satellite communications and integrated sensing and communications systems [45].

From this received signal, the achievable feeder link rate, which dictates the backhaul capacity, is given by the Shannon formula

(4) $$R_{s}=\log_{2}\;\left(1+\frac{|g_{s}^{H}v{|}^{2}}{\sum_{m=1}^{M}|h_{s}^{H}w_{m}{|}^{2}+g_{s}^{H}V_{0}g_{s}+\sigma_{s}^{2}}\right).$$

In (4), $R_{s}$ denotes the achievable rate of the satellite feeder link, and $I_{s}$ represents the total interference-plus-noise power.

The Earth Station decodes the satellite signal and forwards it to the base station via wired backhaul; the base station then transmits downlink symbols to users via beamforming vectors.

At each user equipment *m*, the received signal comprises five components: the intended communication signal from the base station, interference from other users’ signals, leakage from the satellite communication signal, interference from the satellite sensing waveform, and noise. Mathematically,

(5) $$\begin{array}{cc} y_{m}\left(t\right) & =h_{m}^{H}w_{m}\;x_{m}\left(t\right)+\sum_{n\neq m}h_{m}^{H}w_{n}\;x_{n}\left(t\right)\\ & \;+g_{m}^{H}v\;x_{s}\left(t\right)e^{j2\pi f_{s}\left(t\right)t}+g_{m}^{H}s_{0}\left(t\right)+z_{m}\left(t\right). \end{array}$$

where the first term is the desired communication signal for user *m*, the second term represents multiuser interference, the third term is the interference leaked from the satellite communication signal, the fourth term constitutes the interference from the satellite sensing waveform, and the last term is the additive white Gaussian noise at user *m*’s receiver with zero mean and variance $\sigma_{m}^{2}$.

Based on this signal model, the achievable data rate for user *m* is

(6) $$R_{m}=\log_{2}\left(1+\gamma_{m}\right),$$

where $\gamma_{m}$ denotes the signal-to-interference-plus-noise ratio, given by

(7) $$\alpha_{m}\left(W,V,V_{0}\right)=\log_{2}\;\left(g_{m}^{H}Vg_{m}+\sum_{n=1}^{M}h_{m}^{H}W_{n}h_{m}+g_{m}^{H}V_{0}g_{m}+\sigma_{m}^{2}\right).$$

Here, $\alpha_{m}\left(W,V,V_{0}\right)$ represents the logarithmic function of the total received signal-plus-interference-plus-noise power for user *m*, which facilitates the difference-of-convex reformulation of the rate expression.

### 2.2. Satellite Channel Model

The performance of the IST-ISAC system hinges critically on the characteristics of the satellite wireless channel. For low-Earth orbit constellations, the satellite channel encompasses free-space path loss, atmospheric attenuation, Doppler effects, and antenna gain characteristics. The composite channel vector from the satellite to a single-antenna ground receiver is modeled as

(8) $$g=\sqrt{G_{r}}\;\alpha_{\mathrm{FS}}\left(d\right)\;\left(r\;⊙\;b^{1/2}\right),$$

where ⊙ denotes the Hadamard (element-wise) product. In Equation (8), $G_{r}$ is the ground terminal receive antenna gain, $\alpha_{\mathrm{FS}}\left(d\right)$ is the free-space amplitude scaling factor, r is the vector accounting for rain attenuation and phase perturbations, and $b^{1/2}$ is the element-wise square root of the satellite antenna gain vector.

We now detail each component of this model in the order of signal propagation.

1. **Geometric and Free-Space Path Loss:**
  Let $d=\sqrt{d_{h}^{2}+d_{0}^{2}}$ denote the slant range from the low-Earth orbit satellite to a ground receiver, where $d_{h}$ is the satellite altitude (typically 500–2000 km for low-Earth orbit systems) and $d_{0}$ is the ground distance from the sub-satellite point to the receiver. The free-space path loss (Friis law) in linear scale is
  (9) $$L_{\mathrm{FS}}\left(d\right)={\left(\frac{4\pi fd}{c}\right)}^{2},$$
  where *f* is the carrier frequency and *c* is the speed of light. The corresponding field-amplitude factor, which scales the signal amplitude, is
  (10) $$\alpha_{\mathrm{FS}}\left(d\right)=\frac{1}{\sqrt{L_{\mathrm{FS}}\left(d\right)}}=\frac{c}{4\pi fd}.$$
2. **Atmospheric Attenuation:**
  Atmospheric effects introduce additional attenuation. Gaseous absorption is captured by a power factor $A_{\mathrm{gas}}\in \left(0,1\right]$ along the propagation path. Rain attenuation, which varies randomly, is modeled by a power gain β∈(0,1], typically log-normal in decibels
  (11) $$\beta_{\mathrm{dB}}≜20\log_{10}\left(\beta \right)∼N\left(\mu ,\sigma^{2}\right).$$
  Furthermore, we account for mild phase perturbations across the satellite antenna feeds via a vector
  (12) $$r\;≜\;\beta^{1/2}\;\mathrm{diag}\;\left(e^{-j\eta_{1}},\ldots ,e^{-j\eta_{N_{s}}}\right)1\;\in \;C^{N_{s}\times 1},$$
  where $\left\{\eta_{i}\right\}$ are random phase shifts and 1 is an all-ones vector.
3. **Satellite Antenna Gain:**
  The satellite antenna array provides directional gain. Let $b_{n}$ be the linear power gain of the *n*-th feed toward the receiver, and define $b={\left[b_{1},\ldots ,b_{N_{s}}\right]}^{T}$. For low-Earth orbit satellites, we adopt the ITU-R S.1528 pattern, which provides the antenna gain in dBi as a function of the off-axis angle ϕ. The linear gain is obtained through conversion
  (13) $$b_{n}^{\mathrm{LEO}}=10^{G_{\mathrm{ITU}}\left(\phi_{n}\right)/10},$$
  where $G_{\mathrm{ITU}}\left(\phi_{n}\right)$ is the ITU-R S.1528 gain pattern in dBi for the *n*-th feed at off-axis angle $\phi_{n}$. This pattern accounts for the typical beam characteristics of low-Earth orbit satellite antennas, including the main lobe and sidelobe structure.
4. **Ground Terminal Receive Gain:**
  The ground terminal (Earth Station or user equipment) has a receive antenna gain $G_{r}$. For the Earth Station, which uses a directional antenna, $G_{r}>1$; for the user equipments, which are assumed to have omnidirectional antennas, $G_{r}=1$.

The overall channel vector g in (8) thus incorporates all these effects: free-space path loss, atmospheric attenuation (including rain and phase perturbations), satellite antenna gain, and ground terminal receive gain.

### 2.3. Terrestrial Channel Model

The terrestrial wireless channel is another critical component of the IST-ISAC system. The channel between the base station and a ground receiver (either Earth Station or user equipment) experiences path loss, shadowing, and multipath fading.

The large-scale path loss in decibels at distance *d* is

(14) $${\mathrm{PL}}_{\mathrm{ter}}\left(d\right)=\mathrm{PL}\left(d_{0}\right)+10n\log_{10}\;\left(\frac{d}{d_{0}}\right)+X_{\sigma },\;X_{\sigma }∼N\left(0,\sigma^{2}\right).$$

where $\mathrm{PL}(d_{0})$ is the reference path loss at distance $d_{0}$, *n* is the path loss exponent, and $X_{\sigma }$ represents log-normal shadowing.

The small-scale fading is modeled by a sparse multipath channel (Saleh–Valenzuela model)

(15) $$h=\sqrt{\beta_{\mathrm{ter}}\left(d\right)}\sum_{l=1}^{L}\alpha_{l}\;a_{\mathrm{BS}}\left(\theta_{l}\right),\;\beta_{\mathrm{ter}}\left(d\right)=10^{-{\mathrm{PL}}_{\mathrm{ter}}\left(d\right)/10},$$

where $\beta_{\mathrm{ter}}\left(d\right)$ is the linear-scale path loss, $\alpha_{l}∼\mathrm{CN}\left(0,\sigma_{l}^{2}\right)$ are complex path gains, and $a_{\mathrm{BS}}\left(\theta \right)$ is the base station array steering vector for angle of arrival/departure θ. For a uniform linear array (ULA) with $N_{b}$ elements, this steering vector is

(16) $$a_{\mathrm{BS}}\left(\theta \right)=\frac{1}{\sqrt{N_{b}}}\;{\left[1,\;e^{j\pi \sin\theta },\;\ldots ,\;e^{j\pi (N_{b}-1)\sin\theta }\right]}^{T}.$$

In Equation (16), $N_{b}$ is the number of antenna elements at the base station, and θ is the angle of arrival or departure.

## 3. Perfect-Channel State Information Design via Convex–Concave Procedure

This section develops the joint beamforming and sensing design under perfect channel state information. We formulate a sum-rate maximization problem and construct an efficient algorithm based on convex-optimization techniques to solve the non-convex problem, establishing a performance benchmark for the subsequent robust design.

### 3.1. Optimization Problem

Under perfect channel state information, we aim to maximize the terrestrial sum rate by jointly optimizing the satellite beamforming vector v, the base station beamforming vectors $\left\{w_{m}\right\}$, and the sensing covariance matrix $V_{0}$, subject to per-user quality-of-service requirements, self-backhaul capacity limitations, beampattern specifications, and power constraints.

The joint beamforming design is formulated as

(17a) $$\begin{array}{cc} \max_{v,\left\{w_{m}\right\},V_{0}} & \;\sum_{m=1}^{M}\log_{2}\;\left(1+\gamma_{m}\right),\;V_{0}⪰0 \end{array}$$

(17b) $$\begin{array}{cc} s.t.\; & \log_{2}\;\left(1+\gamma_{m}\right)\;\geq \;r_{0},\;\forall m, \end{array}$$

(17c) $$\begin{array}{cc} \; & \sum_{m=1}^{M}\log_{2}\;\left(1+\gamma_{m}\right)\;\leq \;R_{s}, \end{array}$$

(17d) $$\begin{array}{cc} \; & a^{H}\left(\theta_{\ell }\right)\left(\sum_{m}w_{m}w_{m}^{H}+V_{0}\right)a\left(\theta_{\ell }\right)\geq \Gamma_{\ell },\;\forall \ell , \end{array}$$

(17e) $$\begin{array}{cc} \; & {∥v∥}^{2}+\mathrm{Tr}\left(V_{0}\right)\leq P_{s}, \end{array}$$

(17f) $$\begin{array}{cc} \; & \sum_{m}{∥w_{m}∥}^{2}\leq P_{T}. \end{array}$$

In problem (17), constraints (17b) and (17c) enforce the per-user quality-of-service and self-backhaul capacity limitations, respectively, while constraint (17d) guarantees the sensing performance by imposing a minimum beampattern gain $\Gamma_{\ell }$ at each sensing direction $\theta_{\ell }$. Constraints (17e) and (17f) limit the transmit power at the satellite and base station, respectively, and constraint (17a) ensures the positive semidefiniteness of the sensing covariance matrix.

### 3.2. Proposed Solution Method

Problem (17) is a nonconvex optimization problem due to the nonconvex logarithmic rate expressions and the coupled beamforming variables. In fact, such problems are generally NP-hard, which makes finding the global optimum computationally intractable. To address this challenge, we develop an efficient suboptimal solution approach based on the convex–concave procedure by reformulating the problem into a difference-of-convex form.

We first reformulate the problem via a lifting technique, introducing auxiliary semidefinite variables $W_{m}=w_{m}w_{m}^{H}$ and $V=vv^{H}$. The user rate expression in (6) can then be rewritten as a difference of two concave functions

(18) $$R_{m}=\alpha_{m}\left(W,V,V_{0}\right)-\beta_{m}\left(W,V,V_{0}\right),$$

These auxiliary variables, however, introduce additional nonconvex constraints in the form of rank-one conditions

(19) $$\begin{array}{cc} \mathrm{Rank}(V) & =1\;\mathrm{and}\;V⪰0, \end{array}$$

(20) $$\begin{array}{cc} \mathrm{Rank}(W_{m}) & =1\;\mathrm{and}\;W_{m}⪰0,\;\forall m. \end{array}$$

where $\alpha_{m}\left(W,V,V_{0}\right)$ and $\beta_{m}\left(W,V,V_{0}\right)$ are given respectively by

(21) $$\alpha_{m}\;\left(W,V,V_{0}\right)≜\log_{2}\;\left(g_{m}^{H}Vg_{m}+\sum_{n=1}^{M}h_{m}^{H}W_{n}h_{m}+g_{m}^{H}V_{0}g_{m}+\sigma_{m}^{2}\right).$$

(22) $$\beta_{m}\;\left(W,V,V_{0}\right)≜\log_{2}\;\left(\sum_{n\neq m}^{M}h_{m}^{H}W_{n}h_{m}+g_{m}^{H}Vg_{m}+g_{m}^{H}V_{0}g_{m}+\sigma_{m}^{2}\right).$$

Note that $\alpha_{m}\left(W,V,V_{0}\right)$ and $\beta_{m}\left(W,V,V_{0}\right)$ are concave with respect to $W_{m}$, V, and $V_{0}$.

Similarly, the rate of the backhaul satellite link $R_{s}$ can also be rewritten as a difference-of-concave form

(23) $$R_{s}=\alpha_{s}\left(W,V,V_{0}\right)-\beta_{s}\left(W,V_{0}\right)$$

where $\alpha_{s}\left(W,V,V_{0}\right)$ and $\beta_{s}\left(W,V_{0}\right)$ are represented respectively as

(24) $$\alpha_{s}\;\left(W,V,V_{0}\right)≜\log_{2}\;\left(g_{s}^{H}Vg_{s}+\sum_{m=1}^{M}h_{s}^{H}W_{m}h_{s}+g_{s}^{H}V_{0}g_{s}+\sigma_{s}^{2}\right).$$

(25) $$\beta_{s}\;\left(W,V_{0}\right)≜\log_{2}\;\left(\sum_{m=1}^{M}h_{s}^{H}W_{m}h_{s}+g_{s}^{H}V_{0}g_{s}+\sigma_{s}^{2}\right).$$

After substituting (21), (22), (24), and (25) into problem (17), aggregating the constraints yields the following optimization problem

(26) $$\max_{V⪰0,\;V_{0}⪰0,\;\left\{W_{m}⪰0\right\}}\sum_{m=1}^{M}\left\{\alpha_{m}\left(W,V,V_{0}\right)-\beta_{m}\left(W,V,V_{0}\right)\right\}.$$

subject to

(27) $$h_{m}^{H}W_{m}h_{m}\geq \left(2^{r_{0}}-1\right)\left(\sum_{n\neq m}h_{m}^{H}W_{n}h_{m}+g_{m}^{H}V_{0}g_{m}+g_{m}^{H}Vg_{m}+\sigma_{m}^{2}\right),\;\forall m.$$

(28) $$\alpha_{s}\left(W,V,V_{0}\right)+\sum_{m=1}^{M}\beta_{m}\left(W,V,V_{0}\right)-\beta_{s}\left(W,V_{0}\right)-\sum_{m=1}^{M}\alpha_{m}\left(W,V,V_{0}\right)\geq 0.$$

(29) $$\begin{array}{cc} a^{H}\left(\theta_{\ell }\right)\left(\sum_{m=1}^{M}W_{m}+V_{0}\right)a\left(\theta_{\ell }\right) & \geq \Gamma_{\ell },\;\forall \ell , \end{array}$$

(30) $$\begin{array}{cc} \mathrm{Tr}(V+V_{0}) & \leq P_{s}, \end{array}$$

(31) $$\begin{array}{cc} \sum_{m=1}^{M}\mathrm{Tr}\left(W_{m}\right) & \leq P_{T}, \end{array}$$

(32) $$\begin{array}{c} \mathrm{Rank}\left(V\right)=1,\;\mathrm{Rank}\left(W_{m}\right)=1,\;\forall m. \end{array}$$

Problem (26)–(32) remains nonconvex due to the difference-of-convex structure of the objective function and constraints, as well as the rank-one constraints. We now develop an efficient solution approach based on the convex–concave procedure.

Define

(33) $$I_{m}\left(W,V,V_{0}\right)≜\sum_{n\neq m}h_{m}^{H}W_{n}h_{m}+g_{m}^{H}V_{0}g_{m}+g_{m}^{H}Vg_{m}.$$

(34) $$D_{s}\left(W,V_{0}\right)≜\sum_{m=1}^{M}h_{s}^{H}W_{m}h_{s}+g_{s}^{H}V_{0}g_{s}+\sigma_{s}^{2}.$$

and introduce the first-order approximation of $\log_{2}\left(\cdot \right)$

(35) $$\gamma \left(x,y\right)≜\log_{2}y+\frac{1}{\ln2}\frac{x-y}{y}\;\left(y>0\right).$$

At iteration *k*, we approximate the nonconvex terms as follows

(36) $$\beta_{m}\left(W,V,V_{0}\right)\approx \beta_{m}\left(W,V,V_{0};W^{k},V^{k},V_{0}^{k}\right)=\gamma \;\left(I_{m}\left(W,V,V_{0}\right),I_{m}\left(W^{k},V^{k},V_{0}^{k}\right)\right).$$

(37) $$\beta_{s}\left(W,V_{0}\right)\approx \beta_{s}\left(W,V_{0};W^{k},V_{0}^{k}\right)=\gamma \;\left(D_{s}\left(W,V_{0}\right),D_{s}\left(W^{k},V_{0}^{k}\right)\right).$$

(38) $$\begin{array}{cc} \alpha_{m}\left(W,V,V_{0}\right) & \approx \alpha_{m}\left(W,V,V_{0};W^{k},V^{k},V_{0}^{k}\right)\\ \; & =\gamma \;\left(h_{m}^{H}W_{m}h_{m}+I_{m}\left(W,V,V_{0}\right),\;h_{m}^{H}W_{m}^{k}h_{m}+I_{m}\left(W^{k},V^{k},V_{0}^{k}\right)\right). \end{array}$$

Substituting the linearizations from (36)–(38) yields the convex semidefinite programming problem at iteration *k*

(39) $$\max_{\begin{array}{c} V⪰0,\;V_{0}⪰0,\;\left\{W_{m}⪰0\right\} \end{array}}\sum_{m=1}^{M}\left\{\alpha_{m}\left(W,V,V_{0}\right)-\beta_{m}\left(W,V,V_{0};W^{k},V^{k},V_{0}^{k}\right)\right\}.$$

subject to

(40) $$\alpha_{s}\left(W,V,V_{0}\right)+\sum_{m=1}^{M}\beta_{m}\left(W,V,V_{0}\right)-\beta_{s}\left(W,V_{0};W^{k},V_{0}^{k}\right)-\sum_{m=1}^{M}\alpha_{m}\left(W,V,V_{0};W^{k},V^{k},V_{0}^{k}\right)\geq 0.$$

and constraints (27), (29), (30), and (31).

Note that the major computational complexity of Algorithm 1 is dominated by solving the convex semidefinite programming problem in (40) at each iteration. Using an interior-point method, the computational complexity is on the order of

$$O\;\left({\left(M+1+T\right)}^{4}\max{\left\{N_{b},N_{s}\right\}}^{1/2}\log\;\left(\frac{1}{\delta }\right)\right)$$

where δ denotes the solution accuracy.
**Algorithm 1** DC-Based Joint Beamforming and Sensing Design**Require:** 
${\left\{h_{m},g_{m}\right\}}_{m=1}^{M}$, $h_{s}$, $g_{s}$; $\left\{\sigma_{m}^{2}\right\}$, $\sigma_{s}^{2}$; $r_{0}$; $\left\{\left(\theta_{\ell },\Gamma_{\ell }\right)\right\}$; $P_{s}$, $P_{T}$; ϵ; *K***Ensure:** 
v, $\left\{w_{m}\right\}$, $V_{0}$1:Find a feasible initial point $V^{\left(0\right)}$, $V_{0}^{\left(0\right)}$, $\left\{W_{m}^{\left(0\right)}\right\}$ and set k=02:**while** k<K and ΔR≥ϵ **do**3:    Linearize nonconvex terms at $\left(V^{\left(k\right)},V_{0}^{\left(k\right)},\left\{W_{m}^{\left(k\right)}\right\}\right)$ according to (36)–(38)4:    Solve the convex semidefinite programming problem (39) to obtain $\left(V^{*},V_{0}^{*},\left\{W_{m}^{*}\right\}\right)$5:    Update: $V^{\left(k+1\right)}=V^{*}$, $V_{0}^{\left(k+1\right)}=V_{0}^{*}$, $W_{m}^{\left(k+1\right)}=W_{m}^{*}$6:    Compute the sum rate improvement ΔR7:    k=k+18:**end while**9:Extract v and $\left\{w_{m}\right\}$ as the principal eigenvectors of $V^{\left(k\right)}$ and $\left\{W_{m}^{\left(k\right)}\right\}$

Furthermore, the optimal value of problem (39) is non-decreasing during the iterations of Algorithm 1 due to the first-order Taylor approximation of $\beta_{m}\left(W,V,V_{0}\right)$. Meanwhile, the objective value is upper bounded by the optimal value of the original problem (17). Therefore, the sequence of objective values generated by Algorithm 1 is monotonically non-decreasing and bounded above.

Consequently, the convergence of Algorithm 1 can be guaranteed, and the algorithm converges to a stationary point of the original problem.

### 3.3. Convergence of the Proposed Algorithm

The proposed algorithm in Section 3 is based on the convex–concave procedure (CCP), which iteratively solves a sequence of convex semidefinite programming problems obtained by linearizing the non-convex components of the original optimization problem.

Specifically, the achievable rate expressions are written in a difference-of-concave form as

(41) $$R_{m}=\alpha_{m}\left(W,V,V_{0}\right)-\beta_{m}\left(W,V,V_{0}\right),$$

where both $\alpha_{m}\left(\cdot \right)$ and $\beta_{m}\left(\cdot \right)$ are concave functions with respect to the optimization variables.

At iteration *k*, the concave term $\beta_{m}\left(\cdot \right)$ is approximated by its first-order Taylor expansion around the point $\left(W^{\left(k\right)},V^{\left(k\right)},V_{0}^{\left(k\right)}\right)$, yielding a global upper bound

(42) $$\beta_{m}\left(W,V,V_{0}\right)\leq {\tilde{\beta }}_{m}\left(W,V,V_{0};W^{\left(k\right)},V^{\left(k\right)},V_{0}^{\left(k\right)}\right).$$

Let $R^{\left(k\right)}$ denote the objective value obtained at iteration *k*. The following inequality holds

(43) $$R^{\left(k+1\right)}\geq R^{\left(k\right)}.$$

Therefore, the objective sequence is monotonically non-decreasing. Since the objective is also upper bounded due to transmit power constraints and backhaul capacity constraints, the sequence converges to a stationary point.

## 4. Robust Design Under Imperfect Channel State Information

This section develops a robust beamforming algorithm for the practical scenario with imperfect channel state information. Building on the perfect-CSI framework in Section 3, we address the channel uncertainties arising from estimation errors, feedback delays, quantization, and time-varying effects in low-Earth orbit links. A deterministic norm-bounded uncertainty model is adopted to characterize these imperfections, based on which a worst-case robust optimization problem is formulated. The problem aims to maximize the terrestrial sum rate while guaranteeing all quality-of-service, sensing, backhaul, and power constraints under any admissible channel realization. An efficient iterative algorithm is then constructed by integrating the S-procedure with successive convex approximation, jointly optimizing the communication beamformers and sensing covariance matrix $V_{0}$ while managing cross-tier interference. The resulting robust design is evaluated via simulations in Section 5.

### 4.1. Problem Formulation

To capture the practical channel uncertainties in integrated satellite–terrestrial networks, we adopt a deterministic norm-bounded uncertainty model that provides a tractable yet conservative characterization of estimation errors. This model assumes that the true channel vectors lie within an ellipsoidal region around their estimated counterparts, with the radius determined by the maximum expected error magnitude. Such a formulation avoids overly optimistic performance guarantees while remaining amenable to convex reformulation techniques.

Mathematically, the uncertain channels are expressed as:

(44) $$g_{s}={\tilde{g}}_{s}+\Delta g_{s},\;∥\Delta g_{s}∥\leq \varepsilon_{s},$$

(45) $$g_{m}={\tilde{g}}_{m}+\Delta g_{m},\;∥\Delta g_{m}∥\leq \varepsilon_{m},\;\forall m,$$

(46) $$h_{s}={\tilde{h}}_{s}+\Delta h_{s},\;∥\Delta h_{s}∥\leq \delta_{s},$$

(47) $$h_{m}={\tilde{h}}_{m}+\Delta h_{m},\;∥\Delta h_{m}∥\leq \delta_{m},\;\forall m,$$

where ${\tilde{g}}_{s}$, ${\tilde{g}}_{m}$, ${\tilde{h}}_{s}$, and ${\tilde{h}}_{m}$ denote the estimated channel vectors, while $\Delta g_{s}$, $\Delta g_{m}$, $\Delta h_{s}$, and $\Delta h_{m}$ represent the corresponding estimation errors bounded by $\varepsilon_{s}$, $\varepsilon_{m}$, $\delta_{s}$, and $\delta_{m}$, respectively. This bounded uncertainty model is particularly suitable for satellite–terrestrial systems where channel estimation errors can be statistically characterized through measurement campaigns or theoretical bounds.

In light of the norm-bounded imperfect channel state information model (44)–(47), the robust beamforming design is cast as the following worst-case min–max optimization problem

(48) $$\begin{array}{cc} \; & \max_{v,\{w_{m}\}}\;\min_{\Delta g_{s},\;\Delta h_{s},\;\left\{\Delta g_{m},\Delta h_{m}\right\}}\;\sum_{m=1}^{M}R_{m}\\ \; & \mathrm{subject}\;\mathrm{to}\;(17b),\;(17c),\;(17d),\;(17e),\;(17f),\;(44),\;(45),\;(46),\;(47) \end{array}$$

Problem (48) is a semi-infinite non-convex optimization problem, owing to the worst-case formulation over continuous uncertainty sets and the non-convexity of the objective and constraints. The min–max structure further complicates the design, as it requires the optimization of beamforming vectors against the most adverse channel realizations. To tackle this challenging problem, we develop a tractable solution approach based on the S-procedure and successive convex approximation. First, we convert the infinite number of constraints induced by the uncertainty sets into deterministic linear matrix inequalities via the S-procedure. Then, we employ successive convex approximation to approximate the non-convex logarithmic rate expressions with convex surrogates, yielding a sequence of convex semidefinite programs that can be efficiently solved. This methodology guarantees convergence to a stationary point while ensuring robust performance against channel uncertainties.

### 4.2. Solution Approach

To tackle the challenging min–max formulation, we introduce auxiliary variables *x*, *y*, and $\left\{a_{m},b_{m}\right\}$ for all *m* to decouple the logarithmic rate expressions and explicitly incorporate the sensing covariance $V_{0}$ into the constraints. Furthermore, we introduce non-negative slack variables $s_{y}$, $\left\{s_{a_{m}},s_{b_{m}}\right\}$ to convert the inequality constraints into equality forms. This transformation yields the following equivalent reformulation:

(49) $$\begin{array}{cc} \; & \sum_{m=1}^{M}\;\mathrm{Tr}\left(H_{s}W_{m}\right)+\mathrm{Tr}\left(G_{s}V_{0}\right)+\mathrm{Tr}\left(G_{s}V\right)+\sigma_{s}^{2}=e^{x}, \end{array}$$

(50) $$\begin{array}{cc} \; & \sum_{m=1}^{M}\;\mathrm{Tr}\left(H_{s}W_{m}\right)+\mathrm{Tr}\left(G_{s}V_{0}\right)+\sigma_{s}^{2}+s_{y}=e^{y}, \end{array}$$

(51) $$\begin{array}{cc} \; & \mathrm{Tr}\left(H_{m}W_{m}\right)+\mathrm{Tr}\left(G_{m}V_{0}\right)+\mathrm{Tr}\left(G_{m}V\right)+\sigma_{m}^{2}=e^{a_{m}},\;\forall m, \end{array}$$

(52) $$\begin{array}{cc} \; & \sum_{n\neq m}\mathrm{Tr}\left(H_{m}W_{n}\right)+\mathrm{Tr}\left(G_{m}V_{0}\right)+\mathrm{Tr}\left(G_{m}V\right)+\sigma_{m}^{2}=e^{b_{m}},\;\forall m \end{array}$$

With these slack variables, the robust objective function becomes

(53) $$\max_{V,V_{0},\left\{W_{m}\right\}}\sum_{m=1}^{M}\log_{2}\;\left(e^{a_{m}}-e^{b_{m}}\right),$$

subject to the per-user quality-of-service constraint

(54) $$\log_{2}\;\left(e^{a_{m}}-e^{b_{m}}\right)\;\geq r_{0},\;\forall m,$$

and the backhaul capacity constraint

(55) $$\sum_{m=1}^{M}\log_{2}\;\left(e^{a_{m}}-e^{b_{m}}\right)\;\leq \;\log_{2}\;\left(e^{x}-e^{y}\right).$$

At iteration *k*, we employ first-order upper bounds for the exponential terms to convexify the constraints. The interference-plus-noise term at the Earth Station is approximated by

(56) $$\sum_{m=1}^{M}\;\mathrm{Tr}\left({\tilde{H}}_{s}W_{m}\right)+\mathrm{Tr}\left({\tilde{G}}_{s}V_{0}\right)+\sigma_{s}^{2}\leq e^{y^{\left(k\right)}}\left(y-y^{\left(k\right)}+1\right),$$

and for each user equipment, a bound is introduced to handle the non-convex constraint

(57) $$\mathrm{Tr}\left({\tilde{G}}_{m}V\right)+\mathrm{Tr}\left({\tilde{G}}_{m}V_{0}\right)+\sigma_{m}^{2}\leq e^{b_{m}^{\left(k\right)}}\left(b_{m}-b_{m}^{\left(k\right)}+1\right),\;\forall m.$$

where these two parameters equal the following:

(58) $$y^{\left(k\right)}=\ln\;\left(\sum_{m=1}^{M}\mathrm{Tr}\left({\tilde{H}}_{s}W_{m}^{\left(k\right)}\right)+\mathrm{Tr}\left({\tilde{G}}_{s}V_{0}^{\left(k\right)}\right)+\sigma_{s}^{2}\right),$$

(59) $$b_{m}^{\left(k\right)}=\ln\;\left(\sum_{n\neq m}\mathrm{Tr}\left({\tilde{H}}_{m}W_{n}^{\left(k\right)}\right)+\mathrm{Tr}\left({\tilde{G}}_{m}V_{0}^{\left(k\right)}\right)+\mathrm{Tr}\left({\tilde{G}}_{m}V^{\left(k\right)}\right)+\sigma_{m}^{2}\right).$$

To tackle non-convex constraints involving these uncertain channels, we employ the Cauchy–Schwarz inequality to derive conservative approximations for the quadratic forms. Specifically, for the satellite-to-Earth Station channel, $\mathrm{Tr}\left[G_{s}V\right]$ is approximated with the lower bound

(60) TrGsV=g˜sH+▵gsHVg˜s+▵gs=g˜sHVg˜s+2Re▵gsHVg˜s+▵gsHV▵gs≈g˜sHVg˜s+2Re▵gsHVg˜s≥g˜sHVg˜s−2εsVg˜s,

where $▵g_{s}^{H}V▵g_{s}$ has been neglected since it is small compared with ${\tilde{g}}_{s}^{H}V{\tilde{g}}_{s}$ and 2Re▵gsHVg˜s.

Similarly, the other non-convex terms can be approximated respectively by

(61) $$\begin{array}{cc} \sum_{m=1}^{M}\mathrm{Tr}\left[H_{s}W_{m}\right] & \geq {\tilde{h}}_{s}^{H}\sum_{m=1}^{M}W_{m}{\tilde{h}}_{s}-2\delta_{s}∥\sum_{m=1}^{M}W_{m}{\tilde{h}}_{s}∥, \end{array}$$

(62) $$\begin{array}{cc} \mathrm{Tr}\left[G_{m}V\right] & \geq {\tilde{g}}_{m}^{H}V{\tilde{g}}_{m}-2\varepsilon_{m}∥V{\tilde{g}}_{m}∥,\;\forall m, \end{array}$$

(63) $$\begin{array}{cc} \sum_{n=1}^{M}\mathrm{Tr}\left[H_{m}W_{n}\right] & \geq {\tilde{h}}_{m}^{H}\sum_{n=1}^{M}W_{n}{\tilde{h}}_{m}-2\delta_{m}∥\sum_{n=1}^{M}W_{n}{\tilde{h}}_{m}∥,\;\forall m. \end{array}$$

For the desired signal power at user *m*, we have

(64) $$h_{m}^{H}W_{m}h_{m}\geq {\tilde{h}}_{m}^{H}W_{m}{\tilde{h}}_{m}-2\delta_{m}∥W_{m}{\tilde{h}}_{m}∥,\;\forall m.$$

The interference from satellite to user *m* is upper bounded by

(65) $$g_{m}^{H}Vg_{m}\leq {\tilde{g}}_{m}^{H}V{\tilde{g}}_{m}+2\varepsilon_{m}∥V{\tilde{g}}_{m}∥,\;\forall m.$$

To handle the multi-user interference at each user equipment, we introduce auxiliary variables $p_{m}$ to upper-bound the interference

$$\sum_{n=1,n\neq m}^{M}h_{m}^{H}W_{n}h_{m}\leq p_{m},\;\forall m.$$

A conservative upper bound using Cauchy–Schwarz is

$$p_{m}\geq \sum_{n\neq m}{\tilde{h}}_{m}^{H}W_{n}{\tilde{h}}_{m}+2\delta_{m}∥\sum_{n\neq m}W_{n}{\tilde{h}}_{m}∥.$$

Applying the S-Procedure to our robust constraints, slack variables $q_{m}$, $u_{s}$, and $\mu_{m}$ are introduced, yielding the linear matrix inequalities in (66)–(68), where $y^{\left(k\right)}$ and $b_{m}^{\left(k\right)}$ are fixed values from the previous iteration, as defined in (58) and (59).

(66) $$\begin{array}{cc} q_{m} & \geq 0,\;\left[\begin{array}{cc} q_{m}I_{N_{b}}-\sum_{n=1,\;n\neq m}^{M}W_{n}\; & -\sum_{n=1,\;n\neq m}^{M}W_{n}{\tilde{h}}_{m}\\ -\sum_{n=1,\;n\neq m}^{M}{\tilde{h}}_{m}^{H}W_{n}\; & -q_{m}\delta_{m}^{2}+p_{m}-{\tilde{h}}_{m}^{H}\left(\sum_{n=1,\;n\neq m}^{M}W_{n}\right){\tilde{h}}_{m} \end{array}\right]⪰0,\;\forall m, \end{array}$$

(67) $$\begin{array}{cc} u_{s} & \geq 0,\;\left[\begin{array}{cc} u_{s}I_{N_{b}}-\sum_{m=1}^{M}W_{m}\; & -\sum_{m=1}^{M}W_{m}{\tilde{h}}_{s}\\ -\sum_{m=1}^{M}{\tilde{h}}_{s}^{H}W_{m}\; & -u_{s}\delta_{s}^{2}-\sigma_{s}^{2}-{\tilde{h}}_{s}^{H}\left(\sum_{m=1}^{M}W_{m}\right){\tilde{h}}_{s}+e^{y^{\left(k\right)}}\left(y-y^{\left(k\right)}+1\right) \end{array}\right]⪰0, \end{array}$$

(68) $$\begin{array}{cc} \mu_{m} & \geq 0,\;\left[\begin{array}{cc} \mu_{m}I_{N_{s}}-V\; & -V{\tilde{g}}_{m}\\ -{\tilde{g}}_{m}^{H}V\; & -\mu_{m}\varepsilon_{m}^{2}-\sigma_{m}^{2}-p_{m}+e^{b_{m}^{\left(k\right)}}\left(b_{m}-b_{m}^{\left(k\right)}+1\right)-{\tilde{g}}_{m}^{H}V{\tilde{g}}_{m} \end{array}\right]⪰0,\;\forall m. \end{array}$$

#### 4.2.1. Transformation to Linear Matrix Inequalities via S-Procedure

To incorporate the norm inequalities into the semidefinite programming framework, we employ the S-Procedure. For quadratic functions $f_{i}\left(x\right)=x^{H}A_{i}x+2ℜ\left\{b_{i}^{H}x\right\}+c_{i}$(i=1,2) with $A_{i}=A_{i}^{H}$, the implication $f_{1}\left(x\right)\leq 0\Rightarrow f_{2}\left(x\right)\leq 0$ holds if and only if there exists μ≥0 such that

$$\mu \left[\begin{array}{cc} A_{1} & b_{1}\\ b_{1}^{H} & c_{1} \end{array}\right]-\left[\begin{array}{cc} A_{2} & b_{2}\\ b_{2}^{H} & c_{2} \end{array}\right]⪰0.$$

For the norm-bounded uncertainty ∥Δh∥≤ρ, we set $A_{1}=I$, $b_{1}=0$, $c_{1}=-\rho^{2}$ to represent $f_{1}\left(x\right)={∥\Delta h∥}^{2}-\rho^{2}\leq 0$.

#### 4.2.2. Additional Convex Approximations via Successive Convex Approximation

For the exponential terms arising from rate expressions, sequential convex approximation is applied

(69) $$\begin{array}{cc} \; & \sum_{m=1}^{M}\mathrm{Tr}\left[H_{s}W_{m}\right]+\sigma_{s}^{2}\leq e^{y^{k}}\left(y-y^{k}+1\right), \end{array}$$

(70) $$\begin{array}{cc} \; & p_{m}+\mathrm{Tr}\left[G_{m}V\right]+\sigma_{m}^{2}\leq e^{b_{m}^{k}}\left(b_{m}-b_{m}^{k}+1\right),\;\forall m, \end{array}$$

where $y^{k}$ and $b_{m}^{k}$ are values from the *k*-th iteration, given by

(71) $$\begin{array}{cc} y^{k} & =\ln\;\left(\sum_{m=1}^{M}\mathrm{Tr}\;\left[H_{s}W_{m}^{k}\right]+\sigma_{s}^{2}\right), \end{array}$$

(72) $$\begin{array}{cc} b_{m}^{k} & =\ln\;\left(\sum_{\begin{array}{c} n=1\\ n\neq m \end{array}}^{M}\mathrm{Tr}\;\left[H_{m}W_{n}^{k}\right]+\mathrm{Tr}\;\left[G_{m}V^{k}\right]+\sigma_{m}^{2}\right). \end{array}$$

These conservative bounds and convex approximations enable the formulation of a robust beamforming design as a convex semidefinite programming problem, which can be solved efficiently via interior-point methods.

#### 4.2.3. Convex Robust Subproblem at Iteration-*K*

Combining (49)–(52), (69)–(70), the linear matrix inequalities (66)–(68), and the power constraints, we obtain the convex robust semidefinite programming at iteration *k*. The optimization problem is formulated as

(73) $$\begin{array}{cc} \max_{\begin{array}{c} V⪰0,\;V_{0}⪰0,\;\left\{W_{m}⪰0\right\},\;x,y,\left\{a_{m},b_{m}\right\} \end{array}}\; & \sum_{m=1}^{M}\log_{2}\;\left(e^{a_{m}}-e^{b_{m}}\right) \end{array}$$

(74) $$\begin{array}{c} s.t.\;{\tilde{h}}_{m}^{H}W_{m}{\tilde{h}}_{m}-2\delta_{m}∥W_{m}{\tilde{h}}_{m}∥\geq e^{a_{m}},\;\forall m, \end{array}$$

(75) $$\begin{array}{c} {\tilde{g}}_{m}^{H}V{\tilde{g}}_{m}-2\varepsilon_{m}∥V{\tilde{g}}_{m}∥\geq e^{b_{m}},\;\forall m \end{array}$$

with the additional constraint for the Earth Station

(76) $${\tilde{g}}_{s}^{H}V{\tilde{g}}_{s}-2\varepsilon_{s}∥V{\tilde{g}}_{s}∥-e^{x}+e^{y}+\sigma_{s}^{2}\geq 0,$$

along with constraints (49)–(52), (69)–(70), (66)–(68), and the power constraints. Problem (73) constitutes a convex semidefinite programming problem and can be solved efficiently using interior-point methods.

The main computational complexity of Algorithm 2 is dominated by solving the convex semidefinite programming problem at each iteration. Specifically, the worst-case computational complexity is on the order of

$$O\;\left({\left(4M+5+T\right)}^{4}\max{\left\{N_{b},N_{s}\right\}}^{\frac{1}{2}}\log\;\left(\frac{1}{\delta }\right)\right)$$

where δ denotes the solution accuracy.
**Algorithm 2** Robust Joint Beamforming and Sensing Design under Imperfect CSI**Require:** ${\left\{{\tilde{h}}_{m},{\tilde{g}}_{m}\right\}}_{m=1}^{M}$, ${\tilde{h}}_{s}$, ${\tilde{g}}_{s}$; $\left\{\sigma_{m}^{2}\right\}$, $\sigma_{s}^{2}$; $\left\{\varepsilon_{m},\varepsilon_{s},\delta_{m},\delta_{s}\right\}$; $r_{0}$; $\left\{\left(\theta_{\ell },\Gamma_{\ell }\right)\right\}$; $P_{s}$, $P_{T}$; *K***Ensure:** v, $\left\{w_{m}\right\}$, $V_{0}$1.Find a feasible initial point $V^{\left(0\right)},V_{0}^{\left(0\right)},\left\{W_{m}^{\left(0\right)}\right\}$ and set k=02.**while** k<K and ΔR≥ν **do**3.   Update the approximation parameters $y^{\left(k\right)}$ and $\left\{b_{m}^{\left(k\right)}\right\}$ according to (58) and (59)4.   Construct convex approximations of the exponential constraints using (69) and (70)5.   Apply the S-procedure to transform the robust constraints into LMIs in (66)–(68)6.   Solve the convex semidefinite programming problem (73) to obtain $\left(V^{*},V_{0}^{*},\left\{W_{m}^{*}\right\}\right)$7.   Update$$V^{\left(k+1\right)}=V^{*},\;V_{0}^{\left(k+1\right)}=V_{0}^{*},\;W_{m}^{\left(k+1\right)}=W_{m}^{*}$$8.   Compute the sum rate improvement ΔR9.   k=k+110.Extract v and $\left\{w_{m}\right\}$ as the principal eigenvectors of $V^{\left(k\right)}$ and $\left\{W_{m}^{\left(k\right)}\right\}$

Note that Algorithm 2 can also address the joint beamforming problem with perfect channel state information by setting the bounds of all channel estimation errors to zero. However, Algorithm 2 incurs a higher computational complexity than Algorithm 1 due to the additional linear matrix inequality constraints introduced by the robust design.

### 4.3. Convergence Analysis

For the robust design under imperfect channel state information, the proposed algorithm combines successive convex approximation (SCA) and the S-procedure to transform the original semi-infinite optimization problem into a sequence of convex semidefinite programs.

At each iteration *k*, the nonconvex exponential and logarithmic terms are approximated using first-order convex upper bounds. For example,

(77) $$e^{y}\leq e^{y^{\left(k\right)}}\left(y-y^{\left(k\right)}+1\right).$$

After applying these approximations together with the S-procedure, the robust optimization problem becomes a convex semidefinite program that can be efficiently solved.

Let $R^{\left(k\right)}$ denote the objective value at iteration *k*. The iterative procedure satisfies

(78) $$R^{\left(k+1\right)}\geq R^{\left(k\right)}.$$

Thus, the objective sequence is monotonically non-decreasing. Because the feasible set is bounded by transmit power and sensing constraints, the algorithm converges to a stationary point.

## 5. Numerical Results

In this section, we evaluate the performance of the proposed joint beamforming and sensing design for the integrated satellite–terrestrial integrated sensing and communications system.

We consider a Ka-band multi-beam low-Earth orbit satellite with $N_{s}=7$ beams, operating at $f_{c}=30$ GHz over a bandwidth B=100 MHz, which follow typical configurations used in Ka-band satellite communication systems, as summarized in Table 1. The satellite channel incorporates free-space path loss, atmospheric attenuation, and antenna gain, where the rain attenuation is modeled as a lognormal distribution (μ=−3.125 dB, σ=1.591 dB) according to commonly adopted satellite channel models in the literature. A terrestrial base station equipped with $N_{b}=9$ antennas in a 3×3 uniform planar array serves M=8 single-antenna users uniformly distributed in a 1000×1000 m^2^ area, where the base station is located at the center and the Earth Station is placed at the origin. This configuration follows commonly used assumptions in satellite–terrestrial network simulations. Channel uncertainties are bounded by $\varepsilon_{s}=\varepsilon_{m}=\delta_{s}=\delta_{m}=0.08$, which are adopted to model practical channel estimation errors in integrated satellite–terrestrial systems. The per-user minimum signal-to-interference-plus-noise ratio requirement is $\gamma_{\min}=-2.85$ dB. Key system parameters include: satellite altitude h=1000 km, Earth Station antenna gain $G_{\mathrm{ES}}=37.7$ dBi, maximum beam gain $G_{\max}=52$ dBi, and satellite EIRP $P_{\mathrm{EIRP}}=36.7$ dBW, which follow typical antenna and link budget configurations used in satellite communication systems. The noise power is set to $\sigma^{2}=-94$ dBm, derived from the standard thermal noise model with $N_{0}=-174$ dBm/Hz. The satellite transmit power budget is $P_{s}=30$ dBW and the base station power budget is $P_{T}=0$ dBW. The convergence tolerance is set to $\nu =10^{-5}$, and the maximum number of iterations is K=30.

We compare the proposed algorithms (PA1 for perfect channel state information and PA2 for imperfect channel state information) with three benchmark schemes:

- DAS (Direct Access Scheme): Terrestrial users directly receive signals from the satellite.
- TDMA (Time Division Multiple Access Scheme): The satellite and the base station transmit signals to the Earth Station and terrestrial users in orthogonal time slots, respectively.
- IBS (Independent Beamforming Scheme): The beamforming vectors of the satellite and base station are optimized independently.

**Figure 2:**
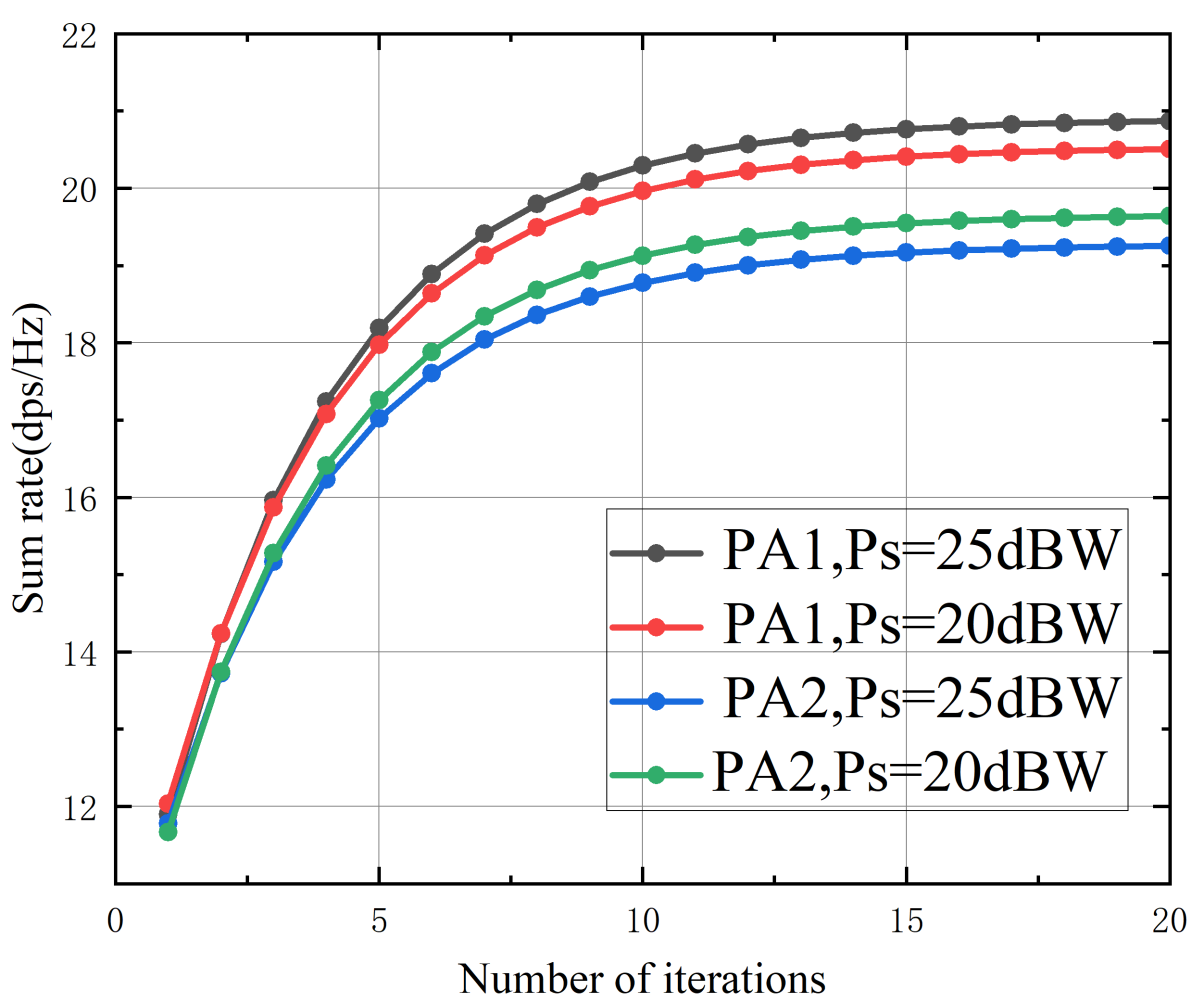
The achievable sum rate versus the number of iterations with $P_{T}=0$ dBW, $N_{b}=3\times 3$, and M=8.

Figure 2 depicts the evolution of the achievable sum rate with respect to the number of iterations for the proposed algorithms PA1 (perfect channel state information) and PA2 (imperfect channel state information). The curves exhibit monotonic growth and stable convergence, with PA1 typically requiring about 20 iterations and PA2 converging within approximately 10 iterations across various satellite power budgets, thereby demonstrating the rapid convergence of the proposed algorithms. The slower convergence of PA1 stems from its lack of conservative margin constraints, necessitating more iterations to refine the beamforming vectors within a less restricted feasible region. Conversely, the uncertainty bounds inherent in the robust design of PA2 impose additional constraints, which effectively structure the feasible region and consequently accelerate convergence. This reliable and efficient convergence behavior corroborates the efficacy of the adopted difference-of-convex reformulation and the subsequent convex–concave procedure, ensuring that the algorithm swiftly attains a high-quality stationary point—an essential attribute for practical implementation in dynamic satellite–terrestrial networks characterized by rapidly varying channels.

**Figure 3:**
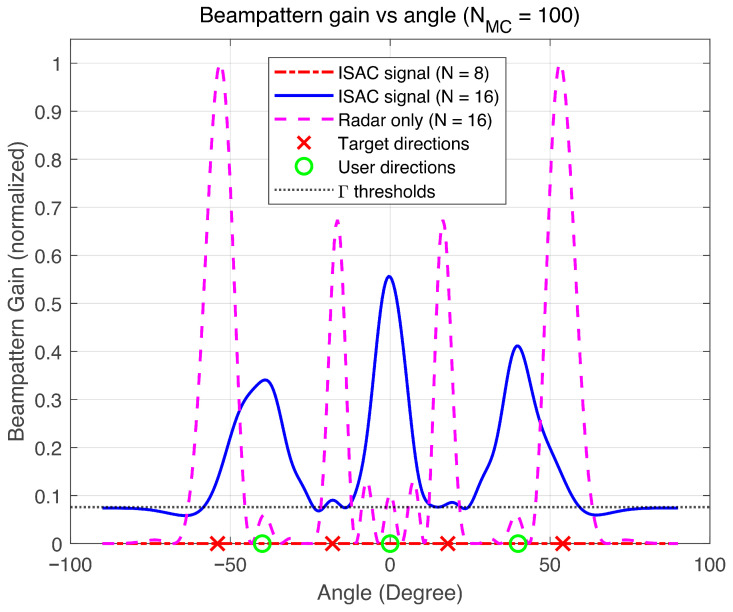
Beampattern gain versus angle.

Figure 3 illustrates the normalized beampattern gain (dB) as a function of the azimuth angle (degrees) generated by the proposed joint integrated sensing and communications design. The radiation pattern displays multiple distinct mainlobes accurately steered towards both communication users (positioned at −30° and 30°) and sensing targets (positioned at −54°, −18°, 18°, and 54°) while simultaneously suppressing sidelobes in other directions. In all target directions, the gain surpasses the predefined threshold Γ, thereby confirming that the specified sensing performance requirements are satisfied. These results validate the efficacy of the proposed joint optimization framework, which concurrently shapes the radiation pattern for multiple communication and sensing tasks through the co-design of communication beamforming vectors and the sensing waveform covariance matrix $V_{0}$. This integrated approach facilitates efficient spectrum sharing and hardware utilization within the integrated satellite–terrestrial sensing and communications architecture.

**Figure 4:**
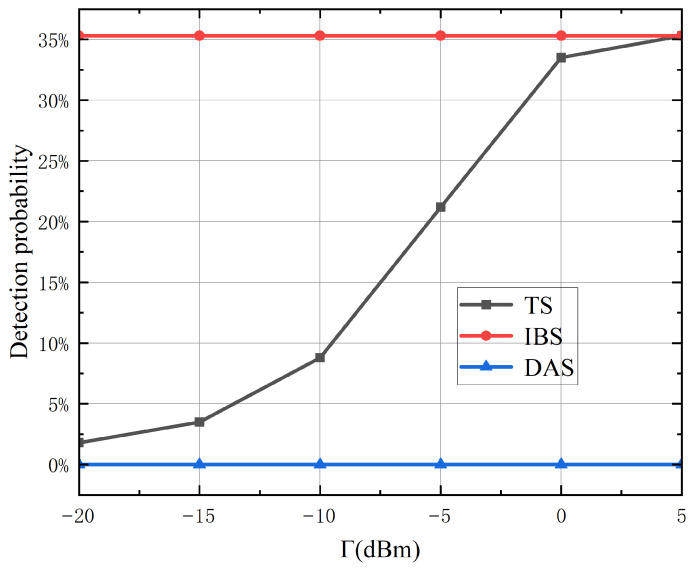
Detection probability versus sensing beampattern threshold Γ.

Figure 4 illustrates the inherent trade-off between the communication sum rate (bps/Hz, horizontal axis) and the radar detection probability (vertical axis) under varying sensing requirement thresholds Γ. As Γ increases, the detection probability exhibits a monotonic improvement, achieved at the expense of a corresponding reduction in the sum rate. This fundamental compromise arises from the allocation of power and degrees of freedom: increasing the resources dedicated to the sensing waveform $s_{0}\left(t\right)$ enhances the signal-to-noise ratio at target directions, thereby improving detection performance, while simultaneously diminishing the resources available for communication signals and consequently reducing data rates. The proposed joint design achieves a Pareto-optimal trade-off curve, demonstrating its ability to flexibly balance the dual functionalities by adaptively adjusting the system’s priority between communication and sensing in accordance with real-time operational requirements.

**Figure 5:**
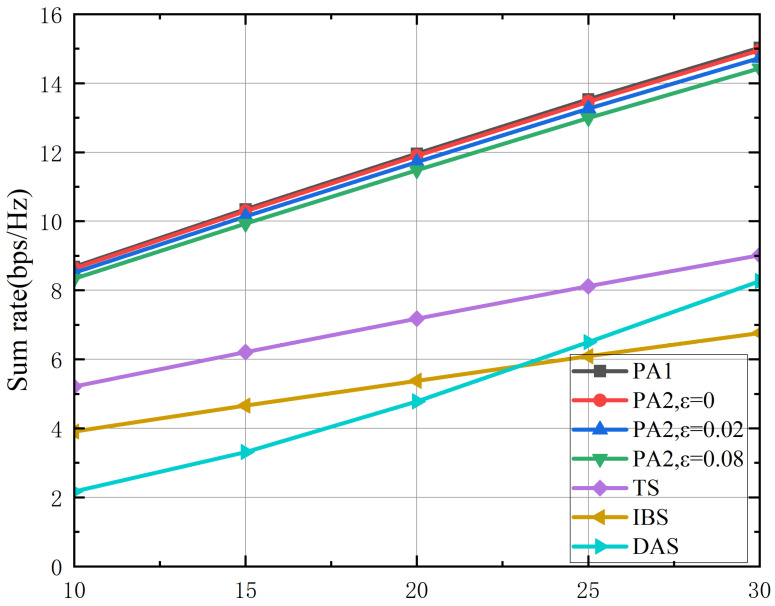
The achievable sum rate versus the power budget of the satellite $P_{s}$ (dBW).

Figure 5 illustrates the relationship between the terrestrial sum rate (bps/Hz, vertical axis) and the satellite transmit power budget (dBW, horizontal axis). As the satellite power increases, the sum rate of all schemes exhibits an increasing trend, with the proposed PA1 (perfect channel state information) and PA2 (imperfect channel state information) algorithms achieving significantly higher sum rates compared to the benchmark schemes. Notably, PA2 performs comparably to PA1 despite channel uncertainty, demonstrating the robustness of the proposed design. This performance superiority originates from the joint beamforming framework, which effectively coordinates satellite and terrestrial transmissions to mitigate mutual interference. The widening performance gap with increasing satellite power indicates that greater power resources enable the joint design to allocate resources more optimally. In contrast, the DAS scheme suffers from severe path loss and unmanaged interference in direct satellite-to-user links, while the TDMA scheme sacrifices spectral efficiency to avoid interference through time division, and the IBS scheme incurs performance degradation due to uncoordinated inter-network interference. Importantly, the sum rate of the proposed algorithms eventually saturates with increasing satellite power, which is attributed to the limited capacity of the satellite-to-Earth Station backhaul link. Once this backhaul becomes the bottleneck, additional satellite power yields no further improvement in the terrestrial sum rate, underscoring the critical role of backhaul constraints in integrated satellite–terrestrial network design.

**Figure 6:**
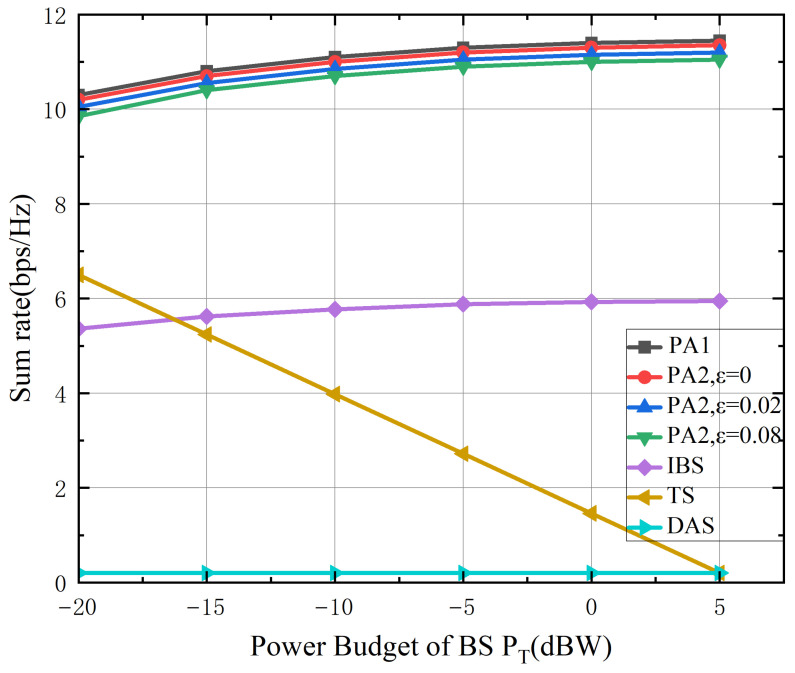
The achievable sum rate versus the power budget of the base station $P_{T}$ (dBW).

Figure 6 depicts the sum rate as a function of the base station transmit power budget (dBW). The sum rate initially increases with base station power due to the enhanced signal-to-interference-plus-noise ratio in the terrestrial access link. However, it eventually saturates as a result of the limited capacity of the satellite-to-Earth Station backhaul link—an inherent characteristic of the ISTS architecture. This saturation demonstrates that the overall system performance is ultimately constrained by the backhaul capacity, underscoring the necessity of provisioning the backhaul to match the potential of the access link in ISTS design. The proposed algorithms consistently outperform all benchmark schemes by jointly optimizing the satellite and terrestrial beamforming under this backhaul constraint, thereby maximizing resource efficiency. In contrast, the benchmark schemes, lacking such coordinated optimization, fail to effectively exploit the increased base station power once the backhaul bottleneck is encountered.

**Figure 7:**
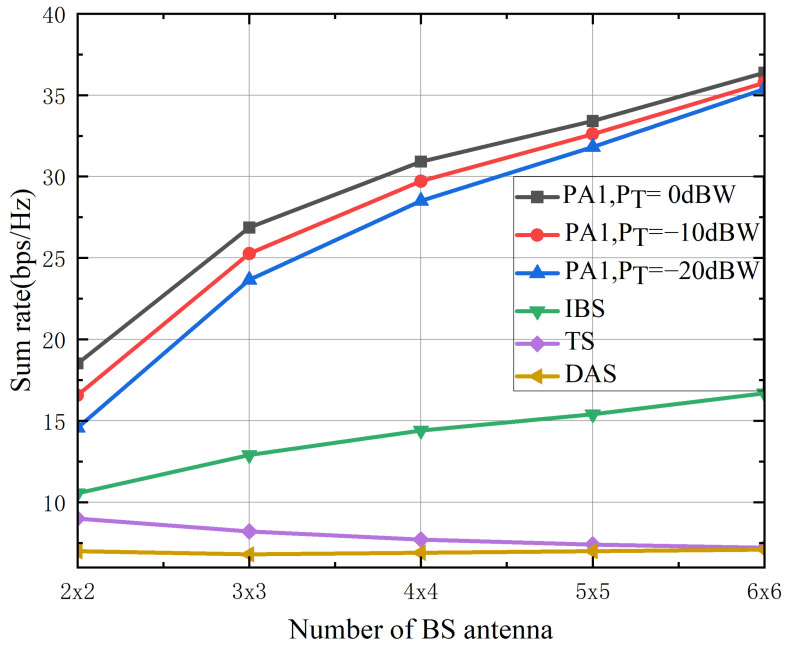
The achievable sum rate versus the number of base station antennas with $P_{s}=30$ dBW and M=3.

Figure 7 illustrates the variation in the sum rate with respect to the number of base station antennas. The sum rate achieved by the proposed PA1 increases with the number of antennas, owing to the enhanced beamforming gain and improved interference suppression, which collectively elevate the signal-to-interference-plus-noise ratio at the terrestrial users. However, this improvement eventually plateaus when the fixed satellite backhaul capacity becomes the bottleneck, indicating that augmenting the number of antennas cannot indefinitely enhance performance in the ISTS architecture. In contrast, the performance of the Independent Beamforming Scheme deteriorates with increasing antennas due to uncoordinated interference toward the satellite link, whereas the time-sharing scheme exhibits moderate gains at the expense of reduced spectral efficiency. These results underscore the existence of an optimal number of base station antennas, beyond which further performance improvements are constrained by the backhaul limitation—a critical consideration in the practical design of ISTS systems.

**Figure 8:**
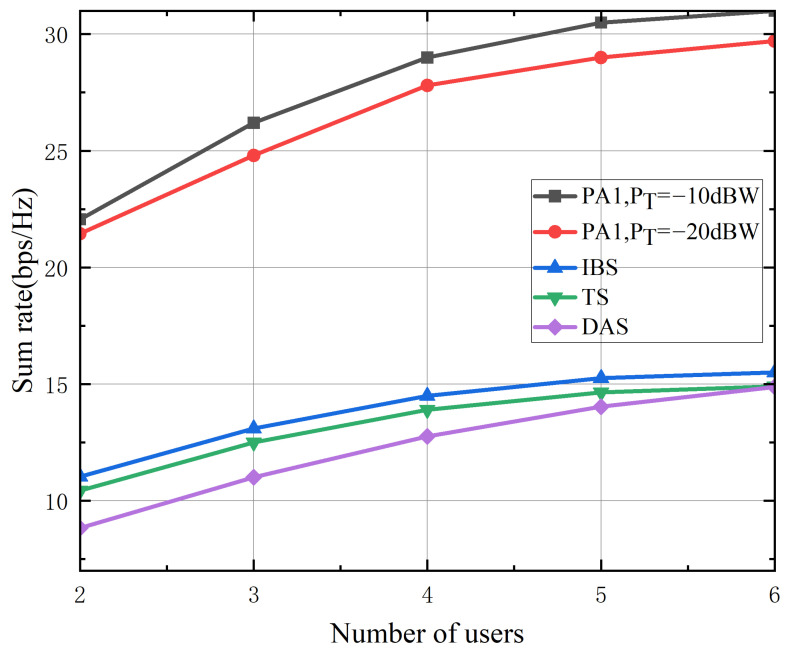
The achievable sum rate versus the number of terrestrial users with $P_{s}=30$ dBW and $N_{b}=4\times 4$.

Figure 8 presents the sum rate as a function of the number of terrestrial users. The sum rate achieved by the proposed PA1 increases with the user count but eventually saturates due to the fixed satellite backhaul capacity constraint, reiterating the performance limitation imposed by the backhaul link. The proposed algorithms consistently achieve the highest sum rate among all considered schemes. The initial increase in sum rate is attributed to multiuser diversity gain, which outweighs the concurrent decrease in per-user rate as the fixed backhaul capacity is shared among a growing number of users. Saturation occurs when the backhaul capacity is fully utilized. The proposed algorithms effectively manage this resource allocation by jointly optimizing the beamforming vectors, thereby maximizing the sum rate while adhering to per-user quality-of-service constraints. In contrast, the benchmark schemes, which lack such joint optimization, achieve lower sum rates. Furthermore, the performance gap between the proposed and benchmark schemes widens with an increasing number of users, underscoring the particular advantage of the joint design in multi-user scenarios characterized by more complex interference management and resource allocation challenges.

## 6. Conclusions

In this paper, we proposed an integrated satellite–terrestrial integrated sensing and communications architecture and formulated a sum-rate maximization problem under practical constraints. For both perfect and imperfect channel state information scenarios, we developed efficient algorithms based on difference-of-convex programming and robust optimization techniques, respectively. Simulation results demonstrate that the proposed joint beamforming and sensing design outperforms benchmark schemes by effectively managing interference and meeting both communication and sensing requirements. The system is shown to be limited by satellite backhaul capacity, while the ISTS-integrated sensing and communications scheme overcomes the severe path loss of direct satellite access for terrestrial users. Furthermore, the design successfully shapes beampatterns for simultaneous communication and sensing tasks, ensuring balanced performance for both functions.

Future work may consider more advanced satellite constellations, dynamic resource allocation, and the integration of artificial intelligence for channel prediction and beamforming design.

## Figures and Tables

**Table 1:** Summary of main simulation parameters.

Parameter	Value
Carrier frequency	20GHz
Number of beams	$N_{s}=7$
Beam diameter	250km
3 dB angle for GEO satellite	0.4°
3 dB angle for NGEO satellite	3.2°
Rain fading	μ=−3.125,σ=1.591
Boltzmann constant	$1.38\times 10^{-23}\;J/K$
BS inter-element spacing	$d_{1}=d_{2}=\lambda /2$
Beam bandwidth	20MHz
Noise temperature	300K

## Data Availability

The original contributions presented in this study are included in the article.
